# SuRVoS: Super-Region Volume Segmentation workbench

**DOI:** 10.1016/j.jsb.2017.02.007

**Published:** 2017-04

**Authors:** Imanol Luengo, Michele C. Darrow, Matthew C. Spink, Ying Sun, Wei Dai, Cynthia Y. He, Wah Chiu, Tony Pridmore, Alun W. Ashton, Elizabeth M.H. Duke, Mark Basham, Andrew P. French

**Affiliations:** a School of Computer Science, University of Nottingham, Jubilee Campus, Nottingham NG8 1BB, United Kingdom; b Diamond Light Source, Harwell Science & Innovation Campus, Didcot OX11 0DE, United Kingdom; c Department of Biological Sciences, National University of Singapore, Singapore 117563, Singapore; d National Center for Macromolecular Imaging, Department of Biochemistry and Molecular Biology, Baylor College of Medicine, Houston, TX 77030, USA; e Department of Cell Biology and Neuroscience, and Center for Integrative Proteomics Research, Rutgers University, NJ 08901, USA

**Keywords:** CCD, Charge-coupled Device, ERF, Extremely Randomized Forest, FIB, Focused Ion Beam, MRF, Markov Random Field, RBF, Radial Basis Function, RF, Random Forest, RoI, Region of Interest, SBF, Serial Block Face, SEM, Scanning Electron Microscopy, SIRT, Simultaneous Iterative Reconstruction Tomography, SLIC, Simple Iterative Linear Clustering, SuRVoS, Super-Region Volume Segmentation, SVM, Support Vector Machines, SXT, Soft X-ray Tomography, TEM, Transmission Electron Microscopy, TV, Total Variation, Interactive segmentation, Hierarchical segmentation, Super-Regions, Semi-supervised learning, Cryo soft X-ray tomography, Cryo electron tomography

## Abstract

Segmentation of biological volumes is a crucial step needed to fully analyse their scientific content. Not having access to convenient tools with which to segment or annotate the data means many biological volumes remain under-utilised. Automatic segmentation of biological volumes is still a very challenging research field, and current methods usually require a large amount of manually-produced training data to deliver a high-quality segmentation. However, the complex appearance of cellular features and the high variance from one sample to another, along with the time-consuming work of manually labelling complete volumes, makes the required training data very scarce or non-existent. Thus, fully automatic approaches are often infeasible for many practical applications. With the aim of unifying the segmentation power of automatic approaches with the user expertise and ability to manually annotate biological samples, we present a new workbench named SuRVoS (*Su*per-*R*egion *Vo*lume *S*egmentation). Within this software, a volume to be segmented is first partitioned into hierarchical segmentation layers (named Super-Regions) and is then interactively segmented with the user's knowledge input in the form of training annotations. SuRVoS first learns from and then extends user inputs to the rest of the volume, while using Super-Regions for quicker and easier segmentation than when using a voxel grid. These benefits are especially noticeable on noisy, low-dose, biological datasets.

## 1 Introduction

Biological imaging techniques have moved from two-dimensional to three-dimensional through tomography, or serial section/imaging in order to provide spatial context, especially for whole cells. For example, in tomography a sample is rotated relative to the beam while 2D projection images are collected at set intervals and later reconstructed into a 3D volume (Lučić et al., 2005). To give contextual information and answer biological research questions, sub-sections of the volume need to be classified according to their biological purpose. In biological volume analysis, data is often represented as a 3D grid of voxels with each voxel in turn representing the absorption of some probe (X-rays or electrons) at that point. Processing of these volumes can be difficult due to their low signal-to-noise ratio and low contrast, in the case of cryo-immobilised samples, and large complex datasets (focused-ion beam or Serial Block Face SEM; Frangakis and Förster, 2004, Lučić et al., 2005, Lučić et al., 2013). This generally requires an expert user manually annotating a large number of voxels.

This time-consuming work can be assisted by classical interactive segmentation methods such as Region Growing (Chang and Li, 1994), Watershed (Vincent and Soille, 1991), Graph Cut (Boykov and Funka-Lea, 2006), Random Walks (Grady, 2006) or Active Contours and Level Sets (Kass et al., 1988, Osher and Sethian, 1988) methods. Recent cryoET and FIB-SEM studies have used various filtering and segmentation methods to ease analysis of their datasets (Prill et al., 2013, Tsai et al., 2014, Vidavsky et al., 2016, Vyas et al., 2016). However, in many cases manual segmentation and basic thresholding methods are still routinely used. Each of these methods has their strengths and weaknesses, and performs differently depending on the properties of individual datasets, but in general, most of them require extensive user intervention to interactively segment a complex cellular volume completely. Region Growing expands a segmentation from a seed point, but will fail to segment organelles if their boundaries are missing or noisy and requires user interaction to correct. Watershed segmentation, which grows multiple regions along the volume simultaneously by flooding from multiple seed points, will face similar problems, with the addition of having difficulties in placing the initial seeds. Graph Cut-based approaches model the segmentation as a graph partitioning problem. They have robust global properties and do not require seeds; however, they do require appearance models for each of the object classes, which are not always obtainable. Finally, Active Contour-based approaches model the shape of the boundaries themselves, and require a manual boundary initialisation around (or inside) each of the objects of interest.

Model or template based searches can be considered a type of segmentation. Recent advances in cross-correlation based template searches have been successfully used to identify protein complexes (Asano et al., 2015, Förster et al., 2010, Liu and Sigworth, 2014), and to semi-automatically segment filaments such as actin or microtubules, and membranes (Rigort et al., 2012). However, in each of these cases, an accurate, correctly scaled, *a priori* model is necessary, limiting their usefulness. In many cases, appropriate structural models can be created using single particle electron microscopy, sub-tomogram averaging, and macromolecular crystallography. However, in the case of polymorphic protein complexes, organelles, etc, models and templates are not appropriate.

Each of the above methods performs very well in specific circumstances. However, biological volumes, particularly near-native state cryo-immobilised datasets, prove challenging due to the necessity of using low-dose imaging conditions and the resulting low signal-to-noise ratio and poor contrast (Frangakis and Förster, 2004, Lučić et al., 2005, Lučić et al., 2013). An additional factor in some 3D biological datasets is the presence of missing wedge artefacts due to data collection over a limited tilt range, resulting in missing information and elongation in the direction of the beam (Frangakis and Förster, 2004, Lučić et al., 2005, Lučić et al., 2013). Each of the above segmentation methods have been extended to try to mitigate some weaknesses (Caselles et al., 1997, Couprie et al., 2011, Li et al., 2010, Veksler, 2008, Vicente et al., 2008, Zhang et al., 2010) however, there is no single framework yet able to solve all of the challenges that biological volumes represent and allow satisfactory semi-automatic segmentation.

More recent methods, such as *ilastik* (Sommer et al., 2011) or the work of Lucchi et al. (2012), use a machine learning-based approach to model the complex properties of the cellular components in biological data. Instead of using standard image processing algorithms to segment the image, the aim is to learn a discriminative model that is then used to segment the rest of the volume. These machine learning approaches have two requirements: (1) available training data (ie. prior expert-segmented volumes) and (2) strong discriminative features extracted from the data. After user annotation, features are extracted and associated with each voxel (such as appearance and textural features) and a classifier is trained to discriminate between the multiple labels. A textural feature is a volumetric filter that enhances an underlying structural feature of the data (e.g. boundaries, edges, patterns). For example, the software package *ilastik* extracts textural features and then uses a Random Forest to classify all voxels in a volume when given user annotations as input (Sommer et al., 2011). The work of Lucchi et al., instead, makes use of supervoxels to reduce the computational complexity and refines the classification of Support Vector Machines (SVM) (Cortes and Vapnik, 1995) with a Markov Random Field for segmentation (Boykov and Kolmogorov, 2004) . This is similar to the method applied with Graph Cuts, but due to the use of SVM, it provides stronger appearance models. This approach not only reduces time and computational complexity, but is also able to extract more discriminative features from supervoxels, which increases classification performance.

Here, we propose a new algorithmic approach, and incorporate it into a workbench. SuRVoS (*Su*per-*R*egion *Vo*lume *S*egmentation) is built based on the fundamental concepts present in the previous two approaches, including the interactive segmentation framework of *ilastik* and the use of supervoxels, unifying the best characteristics from both methods in a single software. SuRVoS first learns from and then extends user inputs to the rest of the volume, while using supervoxels and megavoxels for quicker and easier segmentation than is possible when using a voxel grid. These benefits are especially noticeable on noisy, low-dose, biological datasets. To demonstrate its power we present results obtained using the workbench with two cryogenic soft X-ray tomography (SXT) datasets.

### 1.1 SuRVoS workbench

**Fig. 1:**
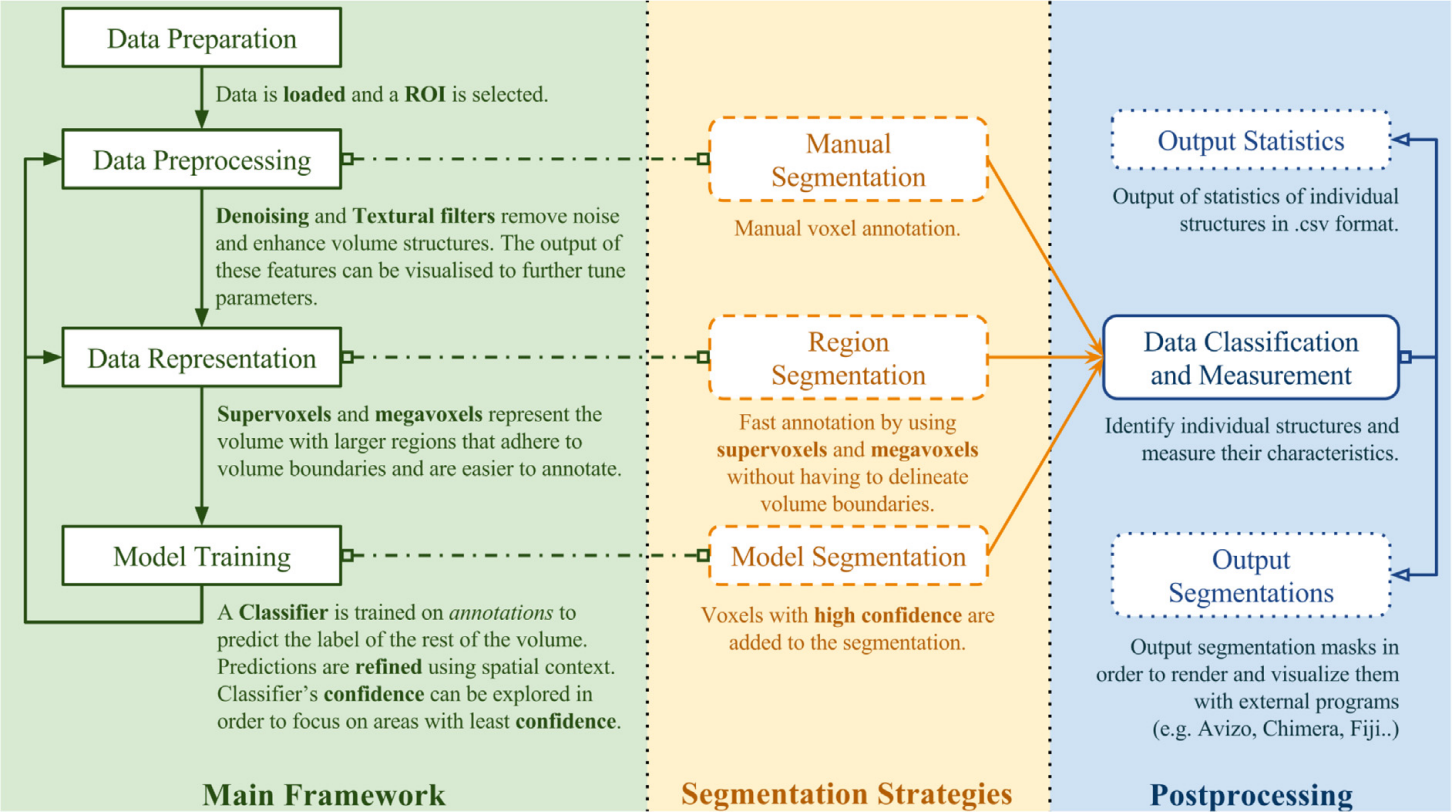
Overview of the SuRVoS framework. Each step has configurable parameters, yet sensible defaults that work well in many cases. The main user workflow (green box), which requires user intervention, consists of the data preparation, preprocessing, representation and model training stages. Segmentation strategies (yellow box) include fully manual, region-based and model-based segmentation. Postprocessing (blue box) consists of data classification and measurement and output of data. Postprocessing steps can be applied to segmentations created by any of the strategies. It is important to note that the user can return to any of the earlier steps at any time to further tune parameters for segmentation of different cellular aspects, or to switch to a different segmentation strategy.

The SuRVoS Workbench combines the human expert's knowledge with data representation, machine learning and active learning techniques. This workbench is designed to semi-automatically segment large biological volumes with the input of a user and to guide the user through the process (Fig. 1). The basic framework of SuRVoS follows these steps:

(1) Data Preparation: Data is first loaded into SuRVoS and a Region of Interest (RoI) is selected.
(2) Data Preprocessing: Denoising filters are used to enhance volume information and remove noise that would reduce the segmentation accuracy. Textural filters are used to enhance relevant features of the volume to better discriminate between different cellular components.
(3) Data Representation: The volume is partitioned into more meaningful regions using hierarchical layers of Super-Regions: voxels, supervoxels (Achanta et al., 2012) and megavoxels (Luengo et al., 2016a).
(4) Model Training: The user defines an arbitrary number of labels and segments a large amount of data with a few clicks using the Super-Region hierarchy. With the user annotated data as input, a classifier is trained and applied to the whole volume in real time. The user can then explore the confidence maps of the classifier, accept voxels with high certainty, and iterate through model training to further refine the results.

At any point the user can go back to any of the previous steps to improve the pre-processing or data representation steps in order to better highlight new target areas. This allows the user to try various parameter configurations and better segment challenging volume regions.

## 2 Materials and methods

### 2.1 SuRVoS requirements

SuRVoS makes extensive use of the HDF5 library (The HDF Group, n.d.) and file format to store intermediate results and features in an efficient on-disk format while reading only the necessary data on-the-fly, which makes SuRVoS well-suited to the exploration of large volumes. Additionally, SuRVoS uses efficient CUDA code to compute most of the features and Super-Regions in parallel. As usual for this type of tool, memory requirements are dependent on the size of the dataset to be analysed. Documentation, a tutorial and the software, including installation instructions and available platforms can be found at https://diamondlightsource.github.io/SuRVoS/ (Luengo et al., 2017).

### 2.2 Sample preparation

Two samples were used as test cases for segmentation with SuRVoS. The first sample was a neuronal-like mammalian cell line (PC-12; Apostol et al., 2003). Briefly, cells were seeded onto gold finder Transmission Electron Microscopy (TEM) grids with holey carbon support (Ted Pella), grown and differentiated, before cryo-immobilization using an EM-GP (Leica) plunge freezer. The second sample was *Trypanosoma brucei* procyclic cells (29–13 strain; (Wirtz et al., 1999). Briefly, cells were cultured and lightly fixed in glutaraldehyde prior to the addition of 200 nm gold fiducials, pipetting onto copper TEM grids with lacey carbon support, and cryo-immobilization using a Mark IV (FEI) plunge freezer.

### 2.3 SXT data collection

Cryo light microscopy (Zeiss AxioImager.M2; Linkam CryoStage CMS196) was used to screen grids for cell distribution, manual handling damage and appropriate ice thickness prior to soft X-ray tomography (SXT) imaging. SXT data were collected on an UltraXRM-S220c microscope (Zeiss Xradia; Duke et al., 2014) using the B24 beamline at 500 eV (2.4 nm wavelength) at Diamond Light Source (B24, n.d.). Images were collected using a 40 nm zone plate, at 812× magnification (16 nm/pixel), on a 1 K by 1 K back-thinned direct detection CCD. Tilt series were collected from ±65° (PC-12 cells) or ±70° (*T. brucei* cells) with a 0.5° step size. Two PC-12 and one *T. brucei* reconstructions were completed using IMOD (Mastronarde, 1997) with SIRT (PC-12 cells) or WBP (*T. brucei* cells) algorithms selected.

## 3 SuRVoS framework

### 3.1 Data preparation

**Fig. 2:**
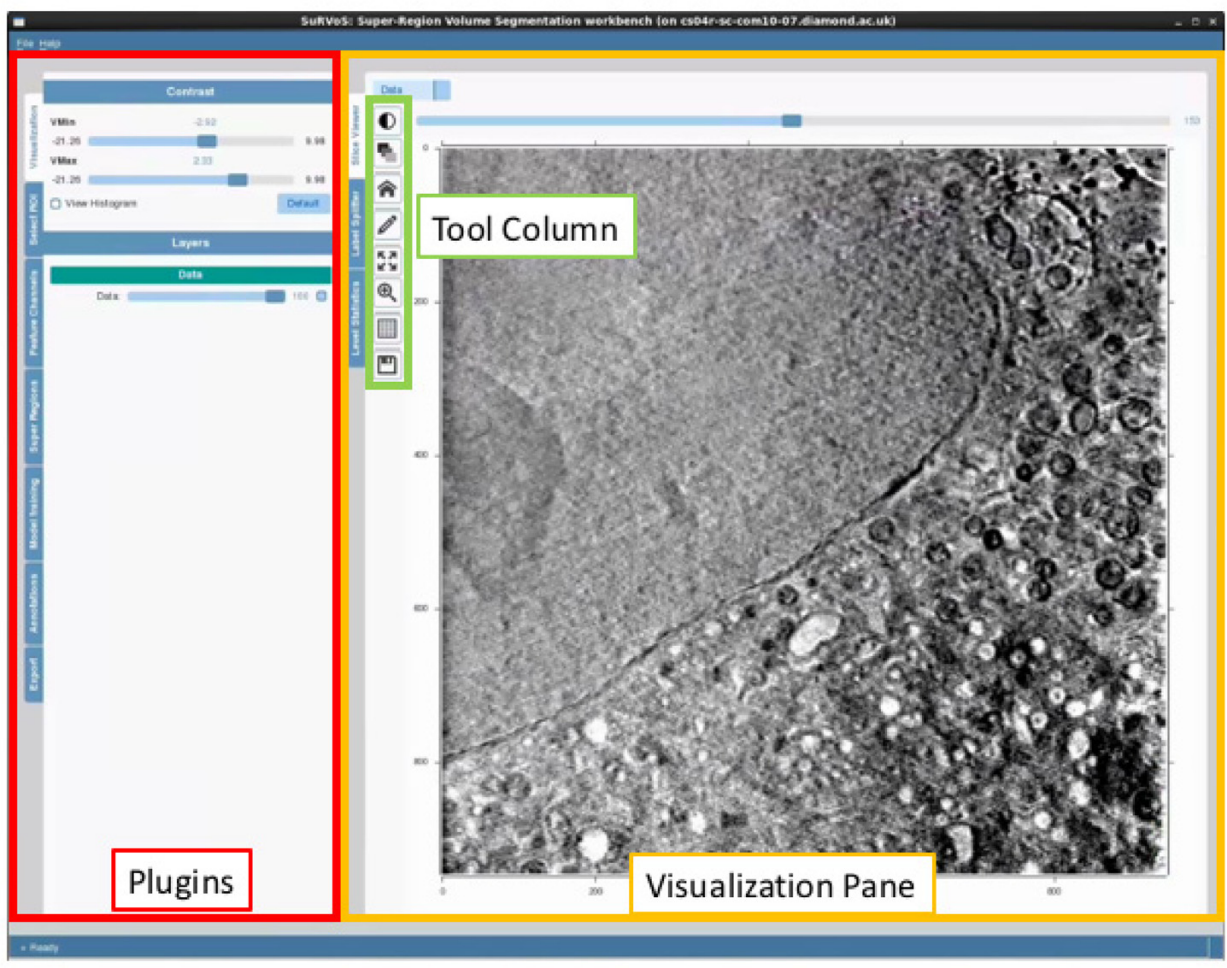
User interface of SuRVoS. The left panel contains the workbench’s main tools, while the right panel contains the visualisation and segmentation workspace. Frequently used tools are also included in an easily accessible column between the panels. Every step during the segmentation workflows (introduced in Section 3.5) outputs a visualisation layer that can be explored to assess the effect of each and to further tune parameters until results are satisfactory.

SuRVoS currently supports the MRC file format (.mrc) and HDF5 file format (.h5, .hdf5). Once data are loaded into the tool, the contrast is automatically selected and can be manually adjusted. The user interface of the workbench includes three separate areas, the Plugins, the Visualisation Pane and the Tool Column (Fig. 2). Parameters can be chosen and applied using the Plugins and assessed in the Visualisation Pane, while the Tool Column houses shortcuts to frequently used tools. Multiple RoIs can be created in the form of bounding cuboids ([zmin, zmax], [ymin, ymax] and [xmin, xmax]). All further actions of the tool can be limited to a designated RoI. This allows the user to select a RoI to test the denoising and textural features in a smaller area before expanding them to the whole volume, or, alternatively, it allows the user to constrain automatic segmentation to a specific RoI.

### 3.2 Data Pre-processing

**Fig. 3:**
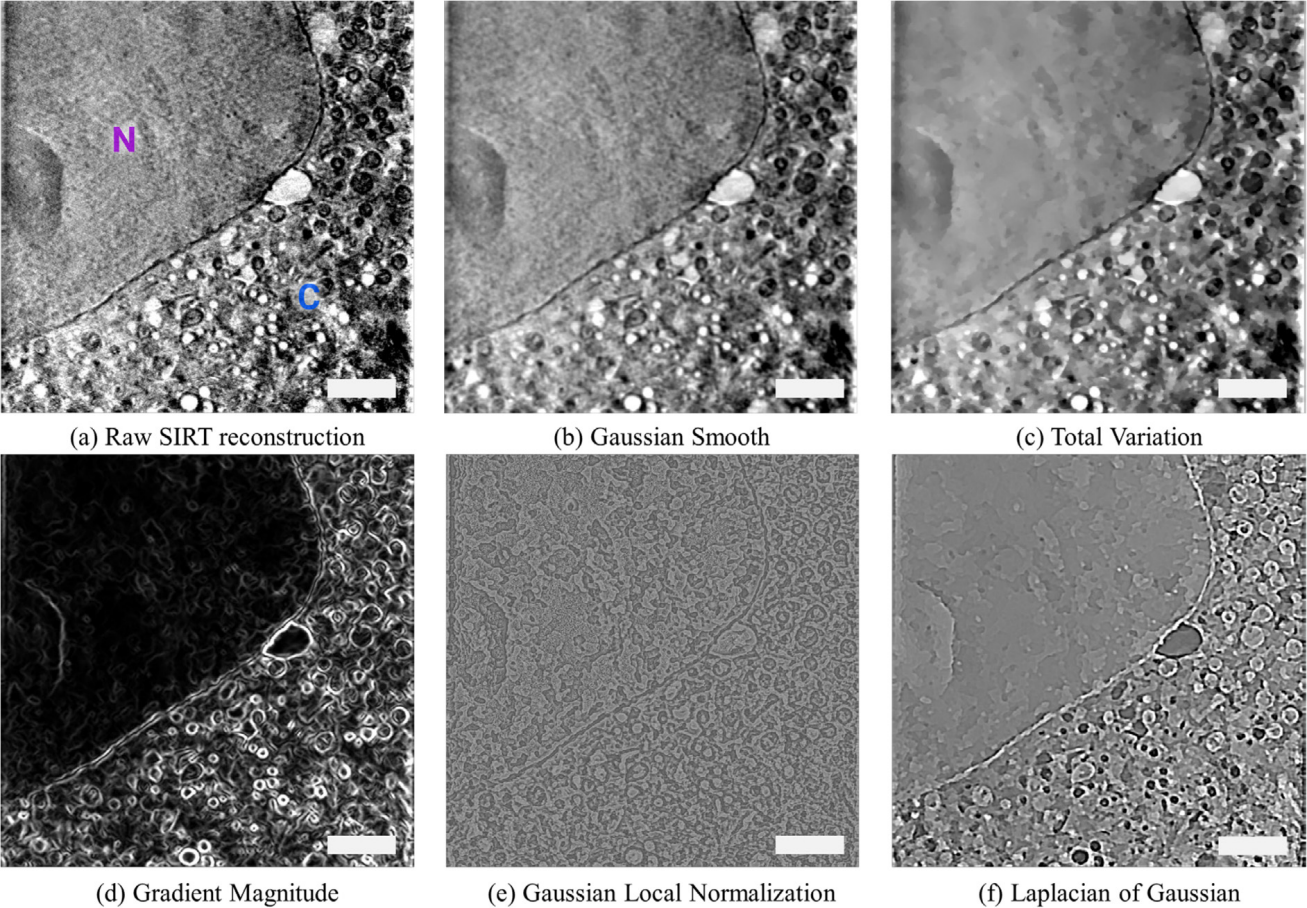
Output of different denoising and textural features on a cryo-SXT dataset. a. A central slice of a SIRT reconstructed tomogram from a PC-12 cell showing a large region of nucleus (marked by N) and a crowded cytoplasm (marked by C), with many, various organelles present. b–c: The same central slice was used to illustrate two different denoising effects. Both Gaussian filter and Total Variation denoising reduce noise, however, the former can over-smooth the image while the latter gives piece-wise constant regions and preserves strong volume boundaries. d–f: Finally, the same central slice was used to illustrate different textural filters, which enhance various elements of the volume. Note: All filtering and denoising methods are applied in 3D. Scale bars are 2.5 μm.

The data pre-processing step combines both denoising and textural feature extraction methods to enhance aspects of the data and make future classification easier. Denoising includes standard Gaussian and Total Variation (TV) filters (Chambolle, 2004, Goldstein and Osher, 2009). While Gaussian filters usually produce over-smoothed results, TV methods preserve strong volume edges and tend to split the volume in piece-wise smooth regions (Fig. 3). Therefore, a Gaussian-denoised volume is better suited for Super-Region extraction, while TV denoising offers better performance in extracting features for classification.

In addition to these basic filters, Gaussian derivative filters, difference of Gaussians and Laplacian of Gaussians, rotation and scale invariant filters and the eigenvalues of the Hessian matrix and Structure tensor are also available as they are more robust feature extraction methods that help identify hidden characteristics of the data. Every filter and feature extracted in this way is represented as a channel of the image, and the user can explore and visualize any channel of the 3D volume through the SuRVoS interface. This helps the user to understand what aspect of the data each of the features enhances, which parameters to choose for each aspect and whether a filter is useful for a particular dataset or not. For example, a TV filter (Fig.3c) may be more appropriate for defining large regions such as the nucleus from the cytoplasm, while the Gradient Magnitude (Fig.3d) or Laplacian of Gaussian (Fig.3f) filters would be more appropriate for segmenting smaller, more nuanced regions such as organelles.

### 3.3 Data representation

**Fig. 4:**
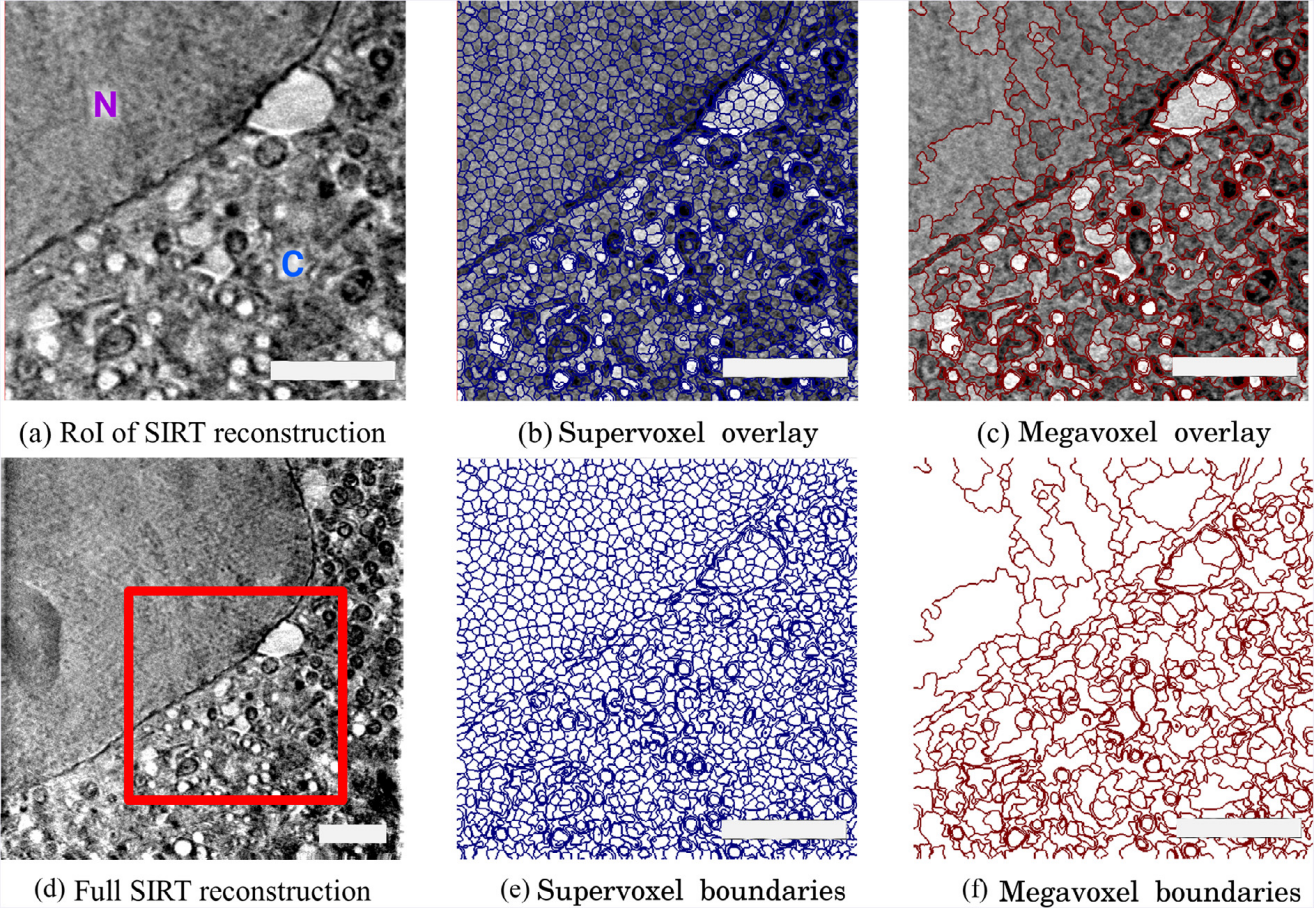
Super-Region hierarchy example using a RoI of a cryo-SXT dataset (PC-12 mammalian cells). A single slice through a full-volume cryoSXT reconstruction with a smaller RoI marked (d, red box). This RoI was chosen to give a clearer view of the Super-Regions while still containing representative areas of both the nucleus and the cytoplasm. The RoI is shown again in (a) and with or without supervoxels (b, e) and megavoxels (c, f) overlaid. Supervoxels are formed by adjacent, similar voxels, while megavoxels group nearby similar supervoxels together. Supervoxels preserve strong image boundaries, in this case delineating the boundary between the nucleus and cytoplasm and many of the boundaries of the organelles within the cytoplasm (b, e). Similarly, megavoxels still preserve boundaries and better represent larger regions (c, f). Note: Both supervoxels and megavoxels are in 3D. Scale bars are 2.5 μm.

Next, the volumetric data is represented as super regions within a 3-layer hierarchical structure. This structure is composed of voxels, supervoxels and megavoxels. Each of these layers is formed by grouping similar, nearby elements of the previous layer. That is, while voxels represent standard volume voxels, supervoxels are groups of adjacent voxels grouped together into a meaningful region that preserve strong volume boundaries (ie. boundaries between different biological features in the image). Similarly, megavoxels are groups of nearby supervoxels that have similar appearance. With this hierarchical partitioning, large areas of the volume belonging to the same object are represented by: a set of thousands of voxels, tens of supervoxels or a few megavoxels (Fig. 4). This hierarchical structure has several advantages compared to the standard voxel grid:

(1) Each of the layers of the hierarchy represents the same volume with many fewer elements than the previous layer, thus, reducing the complexity of annotating or segmenting the volume by several orders of magnitude.
(2) Supervoxels and megavoxels are designed to have a strong boundary adherence and are therefore able to represent the same biological feature without significant loss of information.
(3) The shape and size parameters of the supervoxels are user-definable in order to properly model volumes or areas with different physical characteristics.

SuRVoS implements a custom GPU version of SLIC supervoxels (Achanta et al., 2012) which by default extracts supervoxels of size 10 × 10 × 10 voxels from the volume. This means that the same volume is represented by small groups of around 1000 voxels without significant loss of information (Achanta et al., 2012), as homogeneous regions are represented by single supervoxels. Further larger regions can be extracted as megavoxels (Luengo et al., 2016a, Luengo et al., 2016b). These megavoxels are also extracted in 3D and merge large, similar areas while preserving strong edges and small cellular structures as unique entities. Generally speaking, supervoxels are appropriate for segmenting smaller, more varied features, while megavoxels may be more useful for larger, more similar regions.

### 3.4 Model training

In order to learn from user annotations, voxels or supervoxels need to be described using feature vectors. Features extracted with SuRVoS (Section 3.2**)** are built into discriminative descriptors that are suitable for training. These features form voxel descriptors ***d_p_*** for pixel ***p*** and can be combined into supervoxel descriptors ***s_i_*** by any of the following methods:

(a) Mean of all the feature channels for all the voxels inside each supervoxel.
(b) Histogram of quantised features (using *k*-means) from voxels inside the supervoxel.
(c) Textonized features (using Principal Component Analysis +*k*-means) (Shotton et al., 2007).
(d) Using a SigmaSet (Hong et al., 2009, Luengo et al., 2016a, Luengo et al., 2016b, Luengo et al., 2016c) descriptor, which combines first and second order statistics.

Descriptors can be normalised or standardised as is common in machine learning to boost the performance of the subsequent classifiers. To enhance and add context to supervoxel descriptors, the descriptor of each supervoxel, ***s_i_*** can be extended with the descriptors of the neighbouring supervoxels, denoted as ***N_i,_*** to form a more robust descriptor $\varphi_{i}$ (e.g. a new descriptor is appended to the current supervoxel's descriptor containing the histogram or mean of the features from all the supervoxels that share a boundary with the current one):

$$\varphi_{i}=\left[s_{i}\text{,}\;\frac{1}{\left|N_{i}\right|}\underset{j\in N_{i}}{\sum }s_{j}\right]$$

Once features are extracted, SuRVoS uses machine learning algorithms available through the scikit-learn library (Pedregosa et al., 2011) to classify each Super-Region into experimentally relevant groups. The available classifiers include: Random Forest (RF) (Breiman, 2001), Extremely Random Forest (ERF) (Geurts et al., 2006), Gradient Boosting (Friedman, 2002), Support Vector Machine (SVM) (Cortes and Vapnik, 1995) with different kernels (linear, RBF, etc.) and linear classifiers optimised with Stochastic Gradient Descent (such as linear SVM or Logistic Regression). An experienced user can try different combinations of descriptors and select the most appropriate classifier for each of them. By default, SuRVoS uses an ERF classifier as it requires no parameter tuning and deals well with many kinds of features and normalization.

A selected classifier can be trained on either voxels or supervoxels, the latter being orders of magnitude faster while preserving similar accuracy. The output of the classifier is the label prediction for each of the voxels (or supervoxels) independently. These predictions, however, contain a lot of noise and spurious labels as there is no spatial coherence. To address this, SuRVoS refines the predictions using a Markov Random Field (MRF) formulation, which takes into account neighbouring labels to make the predictions more spatially consistent. Given an undirected graph G=(V,E), where V are the nodes and E is the edge set, and a finite set of labels (or classes) ***C***, the task is to assign the optimal label $c_{p}\in C$ to each p∈V. The general form of a 2nd order MRF enforces unary $\psi_{p}$ and pairwise $\psi_{\mathrm{pq}}$ constraints to the set of nodes and edges,

$$E(c)=\underset{p\in V}{\sum }\psi_{p}(c_{p})+\lambda \underset{p\text{,}q\in E}{\sum }w_{\mathrm{pq}}.\psi_{\mathrm{pq}}(c_{p}\text{,}c_{q})$$

where $c_{p}$ is the label assigned to node p, and $w_{\mathrm{pq}}$ is a similarity weight between nodes p and q. Minimising E(c) yields the optimal labelling $c^{*}$ for the graph G. For the refinement of the segmentation of large volumes, a graph ***G*** is extracted where the nodes are voxels (or supervoxels) and edges connect nearby nodes (in a 6/18/26 neighbourhood for voxels in 3D or supervoxels that are touching, i.e. share boundary voxels). The unary potential is set to the negative log likelihood of the probabilistic output of any of the above classifiers $\psi_{p}(c_{p})=-\log\;p(y|x)$ and the pairwise potentials set as a weighted potts model $\psi_{\mathrm{pq}}=w_{\mathrm{pq}}\cdot [c_{p}=c_{q}]$ where […] evaluates to ***1*** if the inside is true and ***0*** otherwise. The similarity weight between voxels or supervoxels is calculated as a difference between the descriptors of nodes p and q weighted by the amount of boundary voxels they share. This encourages nearby similar nodes to have the same label, smoothing the classifier's result while still preserving the volume's strong boundaries. This is exploiting the property that volume features are usually consistent within a biological structure. Inference of the MRF refinement models is performed by means of the FastPD (Komodakis and Tziritas, 2007, Komodakis et al., 2008) libraries.

**Fig. 5:**
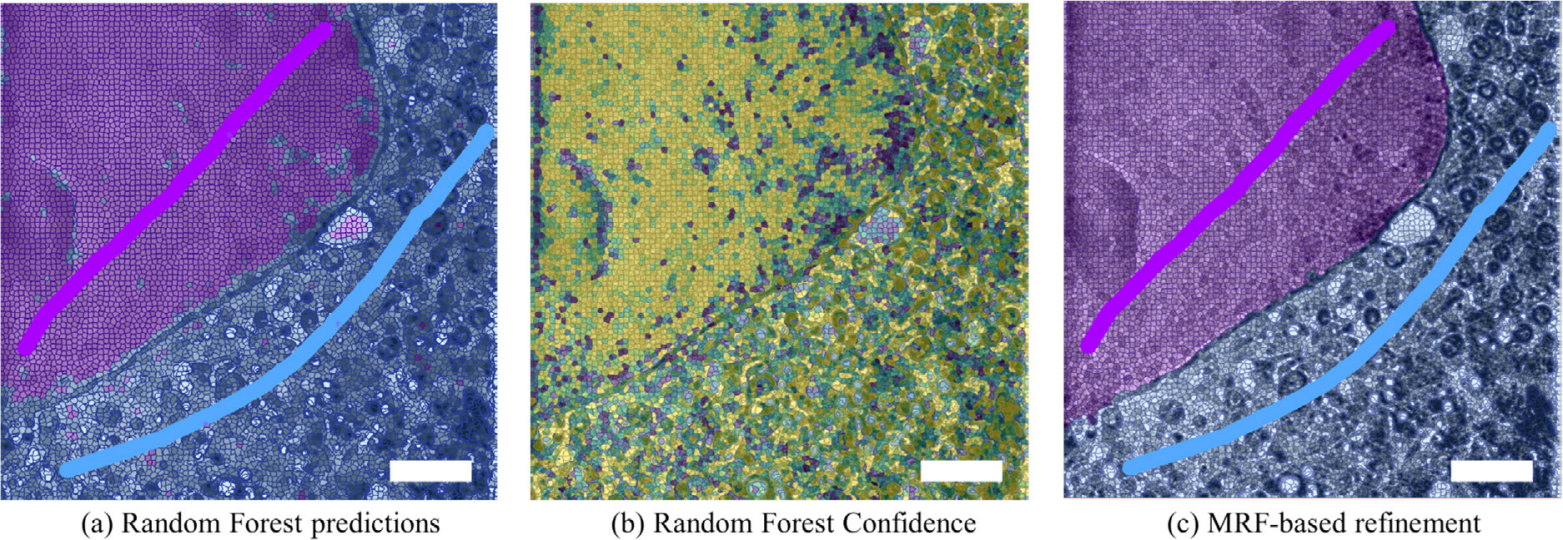
Classification of a representative cryo-SXT volume of a PC-12 mammalian cell into nucleus and cytoplasmic regions. Each image is a central slice of the SIRT-reconstructed volume with supervoxels overlaid. User annotations are shown as elongated lines with no opacity (a, c); purple to denote nucleus and blue for the cytoplasm. (a) A Random Forest (RF) classifier is trained on the user annotations to learn to discriminate between the two regions. (b) The confidence of the RF classifier (yellow for higher and dark blue for lower) can be used to assess the model training. (c) The RF result is refined using a MRF formulation. It is important to note that both classification and refinement are propagated in 3D, and the user annotations shown here are sufficient to accurately segment the central 100 slices of this volume. Scale bars are 2.5 μm.

In practice, this approach allows the segmentation of biological volumes with a reduced level of user interaction. Using the default parameters of the refined classifiers, allowed, in one of the examples presented here, for the segmentation of areas corresponding to the nucleus and cytoplasm with just two brief user annotations (Fig. 5). While this figure is a two-dimensional visualisation, model training using these two user annotations is sufficient to segment the central 100 slices of a 946 × 946 × 350 cryo-SXT volume. By using supervoxels, the classification and refinement steps take just 1–5 s to give predictions for 100 slices of the volume.

The classifier’s confidence can be inspected in order to assess the quality of the classification. Confidence levels can be used as a guidance for further annotations, as annotating uncertain areas (darker areas in Fig.5b) would help the classifier to better understand the data and obtain better performance. Additionally, SuRVoS contains a Confidence Acceptance Tool, which allows the user to save predictions based on the classifier’s confidence level.

### 3.5 User workflows

Applying the user's knowledge to quickly generate training data (in the form of annotations) is a key feature of SuRVoS. The user can annotate volume slices at any of the Super-Region levels: voxels, supervoxels or megavoxels. Thus, with a single manual annotation, all the voxels, supervoxels or megavoxels that the pen tool passes through are assigned to the selected label. This means the user can annotate vast regions of voxels with a minimal amount of effort. Labels can be created as required to correspond to image content and assigned a custom colour and name.

To make the annotation task more intuitive, SuRVoS uses a Segmentation Label Hierarchy. Segmentation labels can be created as needed and newer labels can be placed within parent labels to intelligently restrict model training and the segmentation space. For example, it may be useful to first segment large regions from each other (in one of our examples, nucleus from cytoplasm) before segmenting the nucleoli within the nucleus and the organelles within the cytoplasm. By exploiting this contextual information, which is known to improve the performance of classifiers (Li et al., 2009, Tu and Bai, 2010), individual areas and organelles can be segmented separately in a more efficient manner. In most cases, a two-level label hierarchy is sufficient, separating large volume areas in the first level and assigning smaller objects of interest to the second layer. In SuRVoS, each segmentation label creates a separate annotation mask. This annotation mask can either be directly output, or can be used within SuRVoS to output only the data within the mask for downstream data visualisation purposes.

SuRVoS provides the user with three main workflows with which to segment data: manual segmentation, region-based segmentation, and model-based segmentation. As with other available tools, such as Amira/Avizo (“Avizo,” n.d.), IMOD (Kremer et al., 1996), Fiji (Schindelin et al., 2012) or Chimera (Pettersen et al., 2004), the user can manually annotate the voxels of the volume directly. This approach, at the expense of extensive manual labour, can enhance segmentation in difficult areas where alternative schemes do not achieve the desired level of segmentation accuracy. In the second segmentation approach, using Super-Regions (both supervoxels and megavoxels), different areas of the volume can be rapidly annotated without having to manually delineate a region’s boundaries. As Super-Regions provide good boundary adherence, annotating cell areas is simplified and more time-efficient. Additionally, region annotations can make use of the Segmentation Label Hierarchy to limit a label to a previously defined area of the volume, preventing segmentations from exploding out of an area of interest. The third approach, using descriptors and annotations from previous steps (both manual and region-based annotations), machine learning algorithms can be used to extend annotations to the rest of the volume. This semi-automatic segmentation is aided by a confidence map and can be used to iteratively add to segmentations to train the descriptor. As before, training and prediction of segmentations can be limited to a certain area of the volume by using the Segmentation Label Hierarchy.

### 3.6 Data classification and measurements

**Fig. 6:**
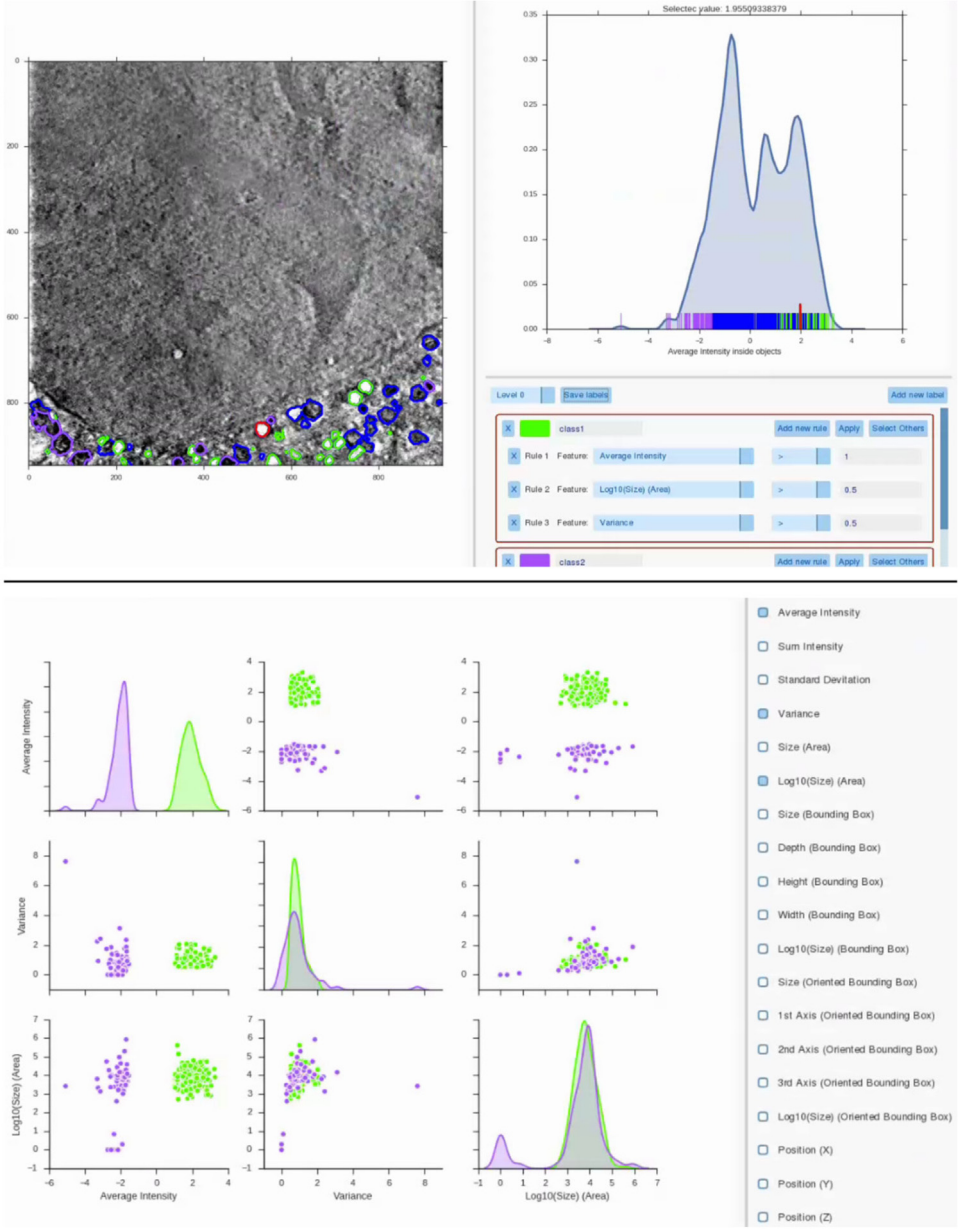
Label Splitter (top) and Label Statistics (bottom) tools allow for classification and analysis of data. After segmentation is complete, the Label Splitter tool (top) can be used to compute appearance, size and shape statistics from each object. Using these statistics, rules can be created to distinguish between object groups (e.g.: green and purple classes). Each object can be selected in the data and found in the plot and vice versa (red object and red line). New object classes can then be made into segmentation labels, and used for comparative analysis in the Label Statistics tool (bottom), where different classes can be visually compared. Results for both tools can be exported as figures or. CSV files for further analysis.

Once the user has segmented the desired structures from the volume, SuRVoS provides two main tools for measurement and analysis (Fig. 6). The Label Splitter tool allows the user to classify groups or individual objects within the segmentation. Using various measures extracted from each object (such as shape, size and average intensity within a selected feature channel) rules can be created to describe a type of object and segregate this type of object into a new class. Once the distinctions are made, results can be saved as new labels. Next, the Label Statistics tool can be used to visualize relationships and output metrics for each object in a label. Plots for each of the metrics extracted for each label allow the user to visually examine the differences between them. Numerical metrics and plots can be exported as .CSV files or images for further analysis or figure preparation.

## 4 Results

### 4.1 PC-12 cell

**Fig. 7:**
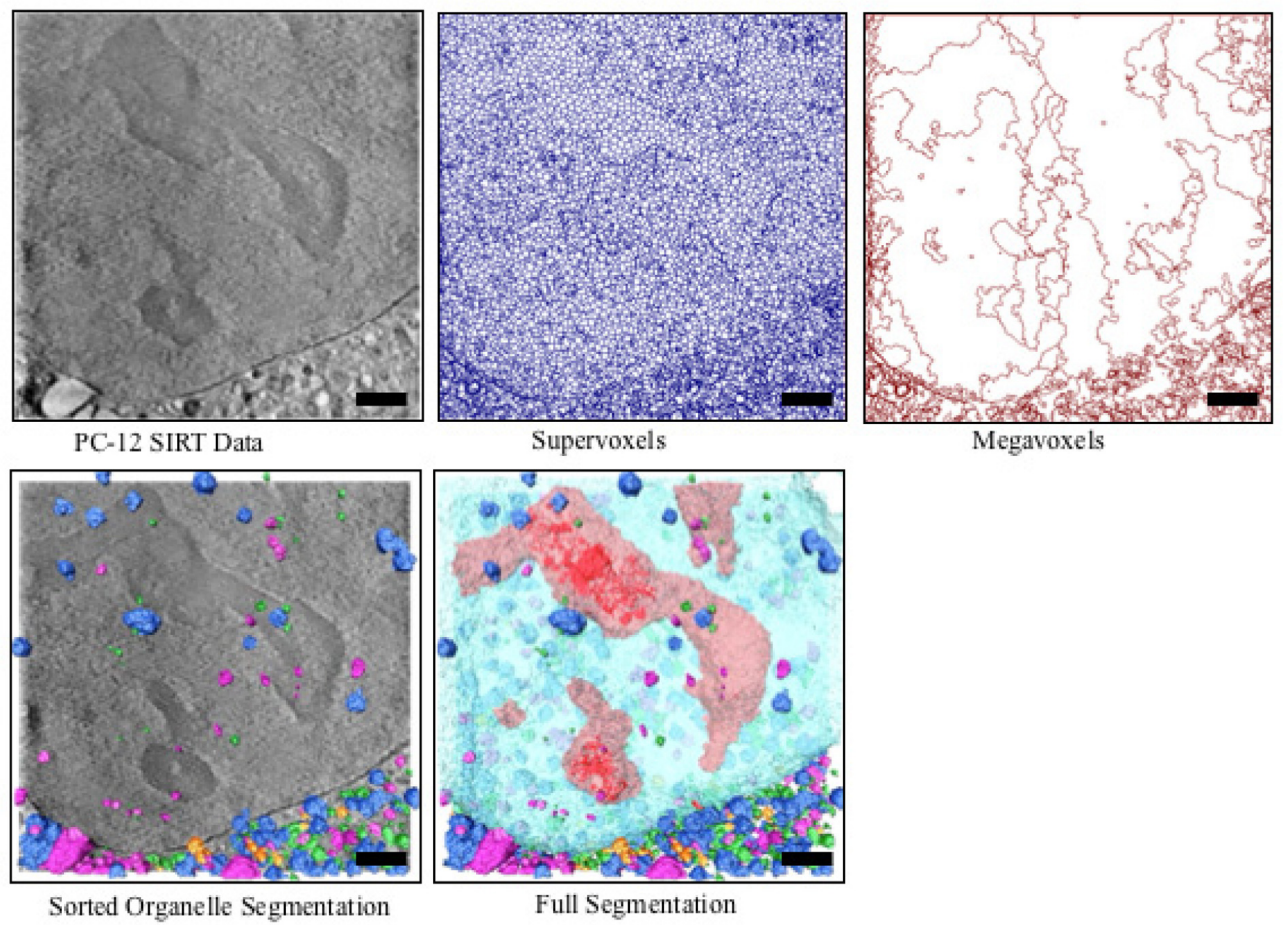
Qualitative results for the PC-12 cell segmentation. Top row shows central slice of the raw data (left) with the corresponding supervoxel (center) and megavoxel (right) extraction. Bottom row shows organelle (left) and full (right) segmentation of the volume respectively. In both segmentations, different classes of organelles, such as mitochondria, lipid droplets and empty vesicles, are represented in various colours. In addition, in the full segmentation, the nucleus is light blue, and nucleoli are red. Scale bars are 2.5 μm.

The 3D data were imported into SuRVoS, where the contrast was adjusted and the volume was clipped to the relevant area. In the pre-processing tab, the data was scaled and appropriate supervoxels and megavoxels were identified (Fig. 7). First, parameters for large, region-defining supervoxels and megavoxels were used in conjunction with model training to segment the nucleus, cytoplasm and extracellular area. Brief manual annotation of a single central slice using supervoxels followed by use of the Confidence Slider and the Area Acceptance tool allowed for the inclusion of voxels that made up the nucleus and cytoplasm through the central slab of the dataset. This process took less than five minutes of user time. It proved more difficult to train SuRVoS to identify the boundaries between the top of the cell and the surrounding ice, and the bottom of the cell as it grew through the supporting holey carbon film. However, when iterating through the same strategy of quick manual annotation, model training, and acceptance of small areas of voxels with high certainty, these boundaries were still identified with minimal manual annotation. The entire region segmentation took approximately 1 h for a 946 pixel by 946 pixel by 311 slices tomogram, representing an approximately 15 μm by 15 μm by 5 μm cellular volume. Next, the supervoxel parameters were tuned to best segment the nucleoli from within the nucleus. Additionally, the Segmentation Label Hierarchy feature was used to ensure only voxels within the nucleus were available for annotation as nucleoli. An iterative model training approach with supervoxels to label the nucleoli and “not nucleoli” was used.

Lastly, appropriate supervoxel parameters for organelle annotation were applied. A higher magnification was used to zoom in on a smaller area of the tomogram and all organelles within that area were annotated approximately every five slices through the 3D volume. Multiple organelle classes were used to ensure separation between near or touching organelles. This rough organelle annotation was repeated across the entire volume, followed by computationally filling holes in each label and smoothing of the segmentation edges using “opening” and “closing” operations.

Next, all organelle classes were used as inputs to the Label Splitter tool. Rules were created based on the average intensity, average variation, average standard deviation and organelle size and location in X/Y/Z. These rules were used to split the organelles into biologically relevant classes such as lipid droplets, empty vesicles and mitochondria (Supplementary Movie 1). Quantitative information, such as number of organelles, voxel number size, and bounding box size, was output for each organelle class.

#### 4.1.1 Trypanosoma brucei cell

**Fig. 8:**
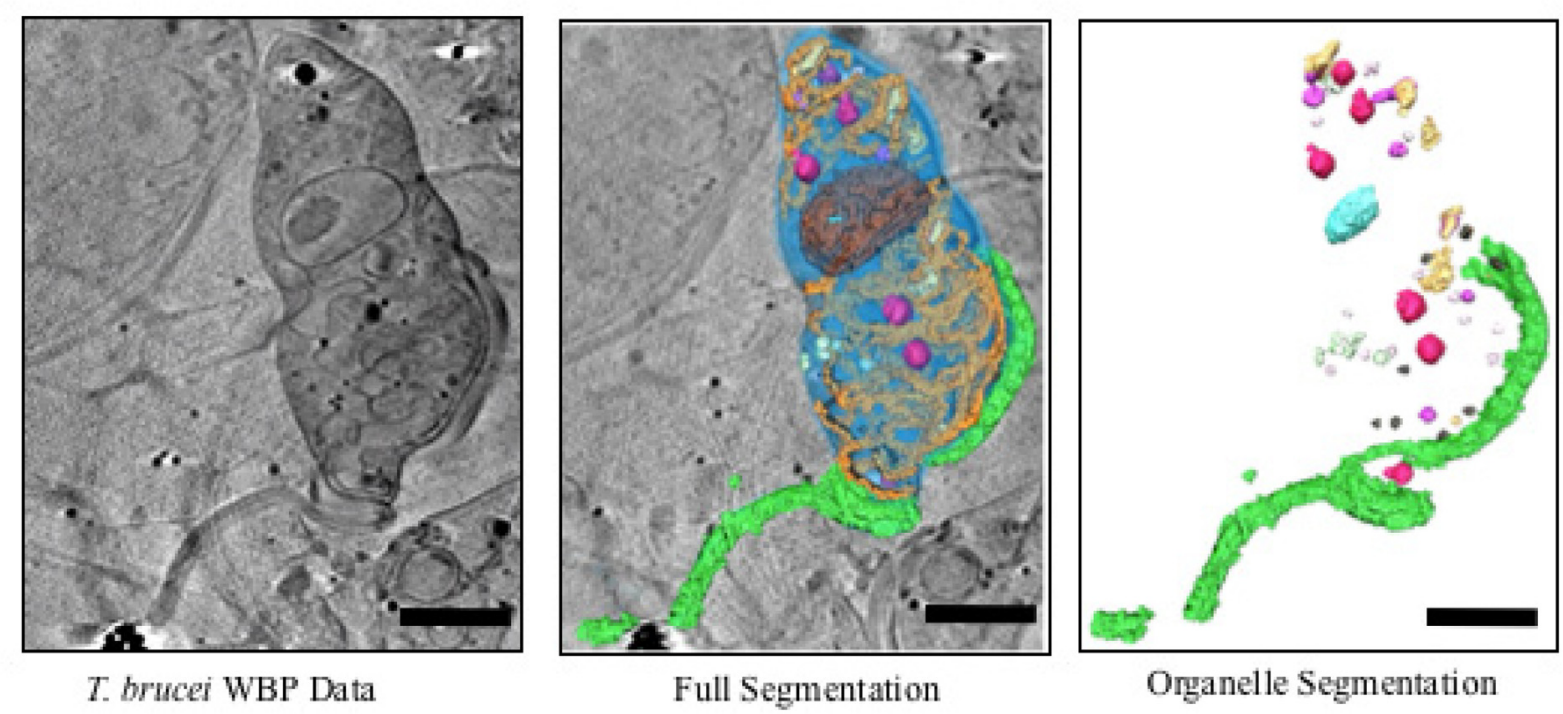
Qualitative results for the Trypanosoma brucei cell. Top row shows central slice of the raw data with the corresponding supervoxel and megavoxel extraction. Bottom row shows organelle and full segmentation of the volume respectively with the flagellum in green, the cell body in blue and organelles in various other colours. Scale bars are 2.5 μm.

The 3D data were imported into SuRVoS, the contrast adjusted and the data clipped to the area of interest. Supervoxels were chosen to allow annotation of multiple types of organelles, while megavoxels were chosen to allow for annotation of the lacey carbon support and cell body of the parasite (Fig. 8).

As before, regions were first defined using megavoxels, however, in this case model training was not used. The lacey carbon support and cell body were segmented using megavoxels; while the parasite’s flagella, nucleus and other organelles were segmented using supervoxels. Computational hole filling and opening and/or closing operations were performed on each segmentation label. In order to segment this dataset, four sets of supervoxel and megavoxel parameters were used and the total segmentation time was approximately eight hours. Classification of organelles was performed by eye, based on the general characteristics of the data (Supplementary Movie 2). Quantitative information about each organelle group was output and used to begin to identify each organelle class.

## 5 Discussion

We present SuRVoS, a new workbench comprising machine learning algorithms and computer vision volume features that uses the inherent features of the data to hierarchically build up regions that respect data boundaries. These regions are then used in conjunction with user interaction to segment 3D data. As features extracted from the data itself are used, SuRVoS is suitable for segmentation of any 3D data. Here, we show examples using soft X-ray cryotomography data, however SuRVoS has been used at a recent training workshop to segment data collected by cryo electron tomography, soft X-ray cryo tomography, focused ion beam Scanning Electron Microscopy (FIB-SEM), and hard X-ray microtomography. Based on these experiences, we believe SuRVoS will be generally applicable to segment volumetric datasets regardless of technique used to collect the data. Additionally, when regions are used to segment the data, the segmentation boundaries are not manually drawn by the user, meaning the resultant segmentation should be less subjective at the voxel level. Additionally, the use of rules based on characteristics of the data paves the way for less subjective identification of organelles within each dataset and between normalised datasets. Further studies would be necessary to fully assess subjectivity in segmentations using SuRVoS Workbench.

There are three ways to use SuRVoS: 1. fully manual voxel segmentation, 2. region-based segmentation (using super- or megavoxels), 3. model-based predictive segmentation. Each of these approaches can be useful depending on the dataset to be segmented and the intended use of the data. In the first approach, segmentation is completed manually without utilising many of the features of SuRVoS, but provides a hand-crafted, expertly derived segmentation. This is essentially equivalent to other available segmentation software. While results will be highly subjective and the process time-consuming (Hecksel et al., 2016) the segmenter may be able to better identify smaller, finer features of interest. The second approach, using supervoxels based on features within the data to segment every few slices, is appropriate for smaller, less uniform features that are unsuitable for model training. The amount of manual segmentation for these features is still high, but because 3D supervoxels are used, meaning user segmentation is only necessary every few slices and without needing to delineate boundaries, the amount of manual segmentation required is still much less than for a fully manual segmentation. The third approach uses the entire suite of features within SuRVoS to quickly train the program and define the boundaries of regions. It can also be used to segment cellular aspects displaying similar characteristics such as similar organelles. This strategy iterates through quick, minimal, manual annotation, followed by model training and acceptance of small areas of voxels with high confidence. The number of voxels that are manually segmented is much smaller in this approach and, compared to a manual segmentation, the time required to segment a volume into various regions is greatly reduced (by a factor of approximately 5). In order to use model training to segment organelles, multiple classes of organelle have to be used such that no organelle is touching another organelle in the same class. This prevents the joining together of two nearby organelles into one object, which would skew classification and statistical output during the label splitting process.

The differences between these three ways of using SuRVoS stem from the supervoxel or megavoxel parameters chosen and the features they are based on. The denoising and textural filters can be stacked and built up, creating filter sets that accentuate specific data characteristics. These filter sets can then be used to create targeted supervoxels or megavoxels for segmentation. Therefore, it is helpful to assess multiple filter, supervoxel and megavoxel options, as these can be changed at any point in the segmentation process. Within the same segmentation, specific parameters can be chosen to segment regions, or to ease segmentation of each specific feature or type of feature within the data. Parameter choice is a critical step in the segmentation process using SuRVoS. Choosing appropriate parameters throughout is necessary for ease and quality of segmentation. Each sample type will be different, and some, like the *Trypanosoma brucei* sample presented here, will benefit greatly from tailored feature, supervoxel or megavoxel parameters.

SuRVoS has been built with a Segmentation Label Hierarchy that allows the assignment of parent and child relationships for labels. Using this feature, segmentation and model training can be restricted to the region of the parent label. Since this hierarchical arrangement constrains the segmentation area, this can speed up computation and further segmentation steps. Template matching tools are not included in SuRVoS, however aspects of the Workbench can ease the use of these tools. For example, SuRVoS can be used to segment large regions to mask away areas where template matching is unnecessary.

The two example segmentations presented here were completed by a biological and segmentation expert (PC-12 cell), and a biological and segmentation novice (*T. brucei* cell). In the case of the PC-12 cell, the segmentation volume was approximately 15 μm by 15 μm by 5 μm (946 pixels by 946 pixels by 311 slices) and took approximately 35 h to segment, classify and visualize fully. For the *T. brucei* cell, the segmentation volume was 11.6 μm by 14.8 μm by 2.2 μm (728 pixels by 927 pixels by 135 slices) and took approximately eight hours to segment fully. The speed-up in segmentation time, in conjunction with the decreased subjectivity of the segmentation method means larger datasets can be more easily analysed in a more quantitative fashion.

## 6 Conclusions

The SuRVoS workbench brings together machine learning models, computer vision techniques and human knowledge within a user interface to interactively segment large 3D volumes. The introduction of Super-Regions (supervoxels and megavoxels) reduces manual segmentation and removes manual delineation of boundaries, potentially decreasing subjectivity. Additionally, SuRVoS provides a wide variety of denoising and textural filters that can be tuned to each sample and used to train a machine learning model to extend the user’s manual segmentations to the rest of the volume. The resulting segmentations can be measured and classified within the tool and exported for further visualisation. In addition to the above benefits, using the features introduced in SuRVoS, user segmentation time is reduced by ∼5× times compared to manual segmentation. Together, these features pave the way for better, more quantitative use of 3D volume datasets in biological sciences.
