# Supplementary figures and images for: SuRVoS: Super-Region Volume Segmentation workbench
Source: J Struct Biol. 2017 Apr;198(1):43–53. doi: 10.1016/j.jsb.2017.02.007 (PMC5405849; doi:10.1016/j.jsb.2017.02.007)

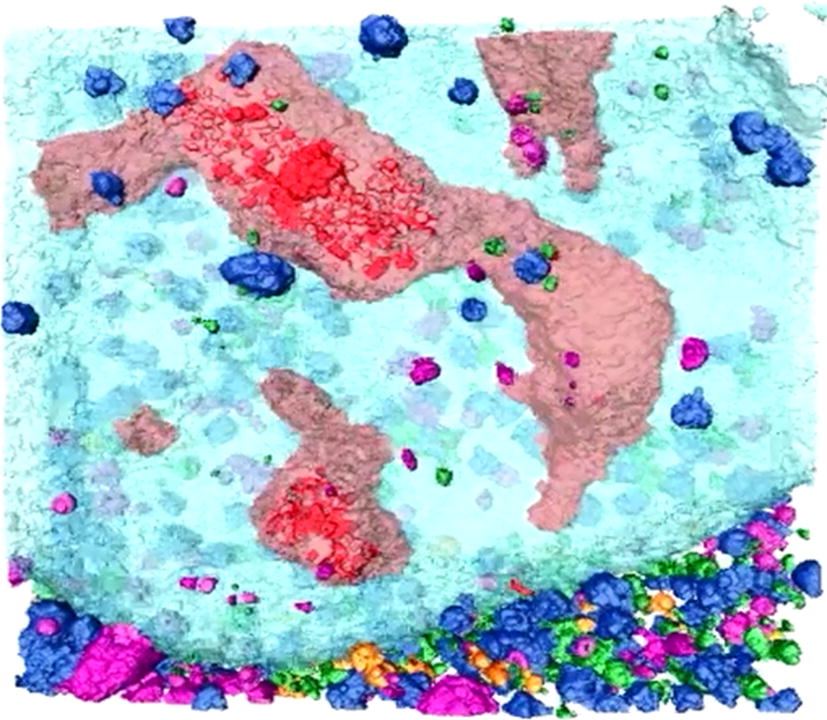

Supplement: Supplementary video 1 [file mmc1.jpg]

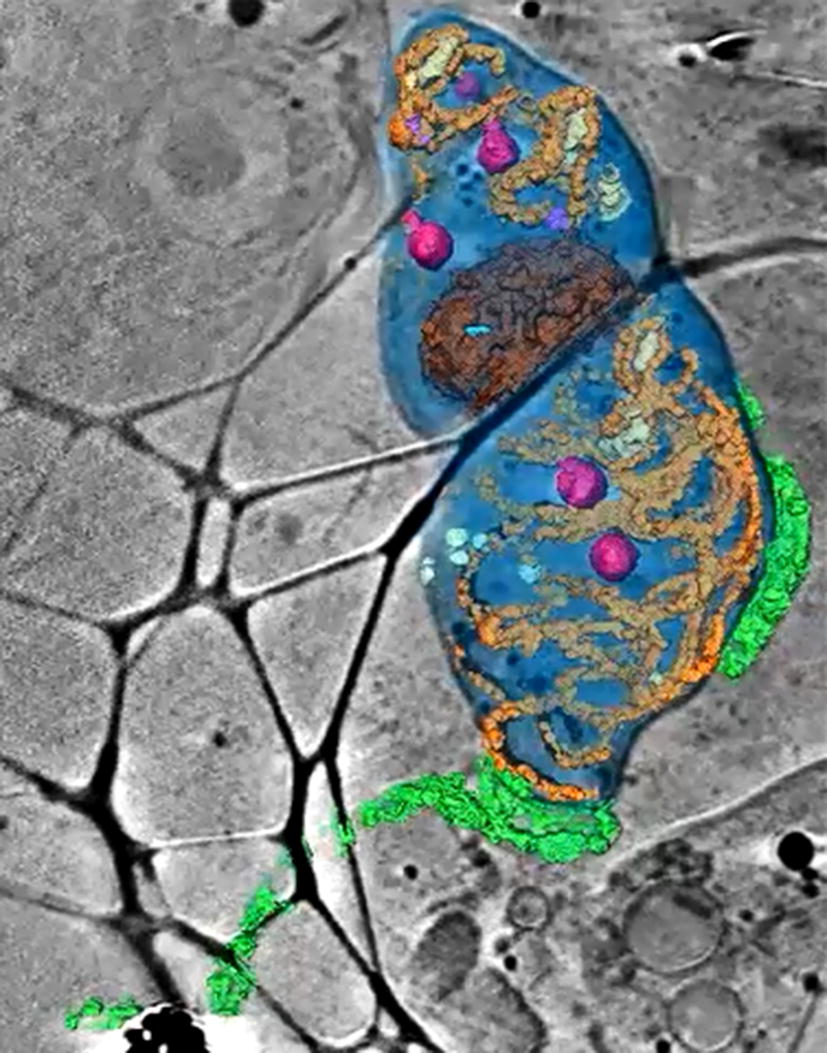

Supplement: Supplementary video 2 [file mmc2.jpg]
